# Muscle Synergy and Musculoskeletal Model-Based Continuous Multi-Dimensional Estimation of Wrist and Hand Motions

**DOI:** 10.1155/2020/5451219

**Published:** 2020-01-28

**Authors:** Yeongdae Kim, Sorawit Stapornchaisit, Hiroyuki Kambara, Natsue Yoshimura, Yasuharu Koike

**Affiliations:** ^1^Department of Information and Communications Engineering, Tokyo Institute of Technology, Yokohama, Japan; ^2^Institute of Innovative Research, Tokyo Institute of Technology, Yokohama, Japan; ^3^PRESTO, JST, Saitama, Japan

## Abstract

In this study, seven-channel electromyography signal-based two-dimensional wrist joint movement estimation with and without handgrip motions was carried out. Electromyography signals were analyzed using the synergy-based linear regression model and musculoskeletal model; they were subsequently compared with respect to single and combined wrist joint movements and handgrip. Using each one of wrist motion and grip trial as a training set, the synergy-based linear regression model exhibited a statistically significant performance with 0.7891 ± 0.0844 Pearson correlation coefficient (*r*) value in two-dimensional wrist motion estimation compared with 0.7608 ±  0.1037 *r* value of the musculoskeletal model. Estimates on the grip force produced 0.8463 ± 0.0503 *r* value with 0.2559 ± 0.1397 normalized root-mean-square error of the wrist motion range. This continuous wrist and handgrip estimation can be considered when electromyography-based multi-dimensional input signals in the prosthesis, virtual interface, and rehabilitation are needed.

## 1. Introduction

Owing to advances in surface electromyography (EMG) signal-based models and algorithms, numerous techniques have been proposed for prosthesis controls and clinical controllers. Though in a discreet fashion, several studies had attempted to convert hand motion into input signals to control prosthetic machines [1], virtual hands [2], and exoskeletons [3], with the aim of estimating both the wrist motion and hand gesture. Nishikawa et al. [1], Sebelius et al. [2], and Kita et al. [4] classified several gestures, such as hand gestures and wrist motions, using algorithms like machine learning, Gaussian mixture models (GMMs), and other linear classifiers (e.g., k-NN and Bayes).

Continuous estimations are applied in response to feedback from real users who require various movements suitable for daily life [5]. Vogel et al. [6] used standard supervised machine learning algorithms to create a mapping between arm/forearm muscle activities and 6-dimensional (6D) position/orientation; this has extended the four rotational degree-of-freedom (DOF) models for the joints of the shoulder and elbow [7]. An algorithm for simultaneous estimation of the three DOFs of the wrist was also proposed [8]; it showed promise of applicability to unilateral amputees by employing a bilateral mirror-training strategy [9]. However, these continuous estimations did not consider the combined motions of the wrist and fingers.

Under the flexor muscles are the multiple finger muscles that lie deep inside the forearm [10]. Many researchers use their expertise to minimize the interference of surface EMG (sEMG), without fully solving crosstalk. For this reason, the sEMG electrode is not free from the inclusion of both external and internal muscle signals. These mixed signals can influence the estimates obtained via the other.

Muscle synergy is defined as a set of muscles recruited by a neural command [11]. A muscle synergy generates a primitive motion, and complex motions are produced by the combination of several synergies [12, 13]. Real-time classification for upper limb motion was conducted using a machine learning technique [14]. However, synergies were differentially weighted according to task constraints [15]; therefore, in this study, two different synergy calculations were attempts: deriving wrist and grip synergies simultaneously and deriving each synergy separately. Besides, synergy model performance with the change of synergy was analyzed, and the choice of number of wrist synergy was checked.

The musculoskeletal model (MSM) is a second-order computational motor control model with nonlinear dynamics. It estimates a one-degree-of-freedom joint angle for flexion and extension considering muscle elasticity and viscosity [16]. Kawase et al. [17] developed a simplified computational model that investigated the estimation of three different joint angles (i.e., elbow, wrist, and finger) with a little influence between finger joint and wrist position estimation.

To realize the prosthetic hand for daily use, wrist motion and grip motion have to be controlled simultaneously; however, few papers treat this problem [17] because of the crosstalk of muscle activation measurement.

This study aims to estimate wrist motion with and without grip motion and compared the estimation performance between optimized MSM and synergy models.

## 2. Materials and Methods

### 2.1. Subjects

Ten healthy subjects (males, aged 28.0 ± 5.7, 9 right-handed, 1 left-handed, none ambidextrous) participated in the experiment. They did not have a history of any form of neurological disorder. They used their dominant hand (either left or right hand) during the conduct of this experiment.

### 2.2. Experimental Protocol

The study protocol was approved by the ethics committee of the Tokyo Institute of Technology (2014042) and was carried out in accordance with the Declaration of Helsinki. Written consent was obtained from each subject before the experiment.

Table 1 indicates the muscle groups chosen to estimate the wrist and grip movements. Five muscles are associated with wrist motion (i.e., ECR, ECU, FCU, FCR, and APL) and two with grip action (i.e., FDS and FDP). Previous wrist-based experiments analyzed ECR, ECU, FCU, and FCR [17, 18], which are the flexor and extensor muscles of the wrist with different deviations (radial and ulnar). In addition to these muscles, in particular APL, an extensor of the thumb was included to trace the radial movement of the wrist. The FDS and FDP—the flexor muscles of the finger—were included to estimate the grip force with a synergy-based model.

**Figure 1:**
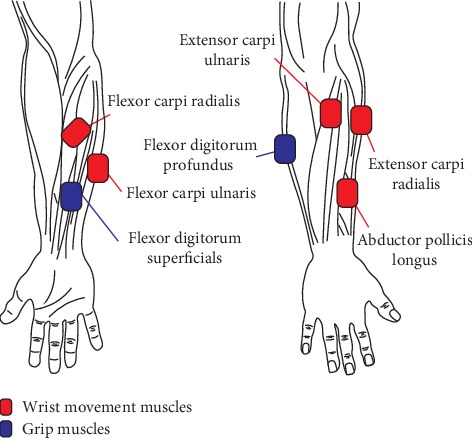
Seven EMG channel placement on five wrist joint-related muscles (red) and two grip muscles (blue).

Figure 1 shows the placement of the EMG sensor on the forearm. The EMG signals were measured using Trigno™ EMG system.

In the experiment, two tasks were conducted. The first task was a wrist motion, which measured motion in different movement conditions. For the second task, isometric grip force was measured in different grip force levels. Thus, the trials were divided into two tasks to check the wrist movement at a certain grip condition and grip force at a certain posture.

**Figure 2:**
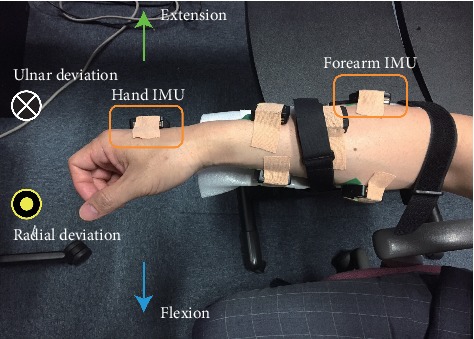
First task: experimental posture in the center-position and movement direction with the placement of the IMU sensors. Yellow-colored rectangles emphasize the positions of the IMU sensors placed at the back of the hand and forearm.

In the first task, EMG was measured using wireless Trigno™ EMG system sensors, and wrist joint angles were measured using the IM sensors of the system.  Figure 2 shows the placement of the IM sensors, which were attached to the back of the hand and the back of the forearm; they were attached to detect the relative wrist joint angle from the forearm.

Subjects placed their forearms on the table fastened by a wrist binder. Thereafter, they performed four wrist motions: flexion, extension, radial deviation, and ulnar deviation. These motions were conducted while the hand was free (no gripping action) and in gripping mode (normal strength). Wrist motions were conducted under three conditions based on the subject's comfort: comfortable maximum limit (with and without grip), half of comfortable maximum limit (without grip only), and stiffened movement with force exertion (without grip only). The subjects performed each motion three times per trial. Three trials were conducted for each condition. Thereafter, the gripping action (without wrist motion) was conducted in the center position (Figure 2) for which the subjects performed strong grips and weak grips. Three trials were conducted for the gripping experiment.

In the second task, EMG and grip force were measured, with the latter done using ReachMAN robot [19]. The subjects adjusted the angle of the grip to their best fit while maintaining a center position posture, as demonstrated in Figure 2, after which the grip force was measured. Three levels of grip strength were performed: strongest, half, and a quarter of gripping power. The strongest grip force (in newton N) varied for every subject with an average of 16.2 ± 3.2 N.

### 2.3. Data Acquisition

The data were sampled separately per signal category using lab streaming layer (LSL) in MATLAB 2018b program base [20]. The EMG signals were sampled at 2000 Hz, IMU sensors at 74 Hz, and ReachMAN force sensor at 100 Hz.

**Figure 3:**
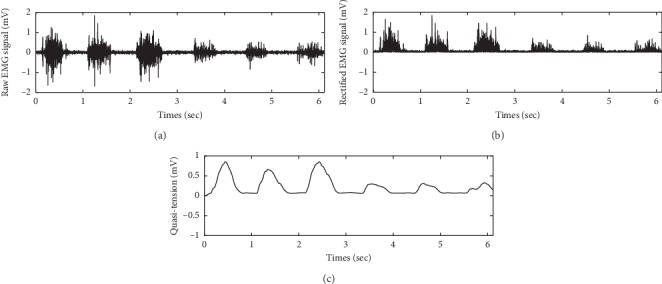
Quasi-tension data filtering process. The low-pass filter was the second-order Butterworth filter with 5 Hz cutoff frequency. (a) Raw EMG signal. (b) Rectified EMG signal. (c) Low-pass filtered EMG signal.

The seven EMG signal channels were filtered and normalized before computing the synergy set. The EMG signals were rectified and filtered using a second-order Butterworth low-pass filtering with 5 Hz cutoff frequency [21].  Figure 3 shows the conversion of an EMG signal. The filtered EMG signals are called “quasi-tension” because it showed a high correlation between the joint torques of its muscle [21].

A recurring issue during experiments and analyses was the fact that the magnitude of EMG signals for each channel had to be changed every time the sensor was detached then attached again. To resolve this, all signals were normalized by the peak activation level of the whole task, including a range of joint angles and maximum effort of trials [22]. Normalization was performed after quasi-tension signal filtering. In this experiment, the combined hand motion tasks, co-activating both grip and wrist motions, were chosen; hence, the normalized quasi-tension signals, which were obtained by filtering and normalizing the EMG signals, were resampled into the other sampling rates and measured together.

### 2.4. Wrist Angle Derivation

The Madgwick IMU algorithm was implemented to estimate the two-dimensional wrist joint angle [23]. IMU sensors were placed on the back of the hand and forearm to track the orientational difference between the hand and forearm.

**Figure 4:**
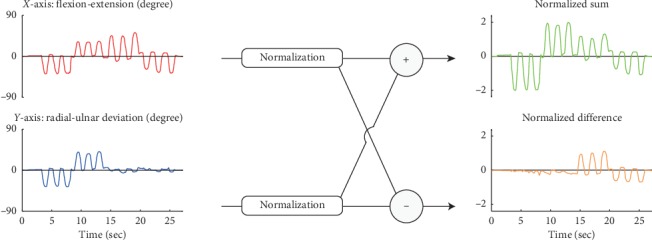
(EPS cross needed) *X*-axis stands for flexion-extension dimension, while *Y*-axis stands for radial-ulnar deviation. To compensate for the inclined diagonal movement of subjects in self-paced movement, two angles were normalized and their sum and difference were subsequently computed.

Subjects performed self-paced movements without visual feedback; consequently, most of them performed diagonal movements even if only vertical and horizontal movements had been requested. To compensate for this, the two angles obtained by the IMU algorithm were normalized by each angle's absolute maximum value; the sum and difference obtained can be seen in Figure 4. In each model, these calculations were estimated and the summation was recalculated to estimate the angle; furthermore, considering the EMG crosstalk error and wide range of wrist angle movements, a comfortable maximum limit trial was mainly used as the train data.

### 2.5. Synergy-Based Linear Regression Model

A synergy-based linear regression model was used to estimate wrist and grip values. To reduce computational costs in a model calculation, a simplified version of the nonnegative matrix method, i.e., the hierarchical alternating least square (HALS) method, was used [24]. Apart from the computational cost, HALS also has a wide capability: it can work with a large number of components [24], in contrast to the canonical NMF method [25], which is only applicable if the number of the sources is greater than the number of components; it can work in conditions where the number of components is large [24]. This feature of HALS is appropriate when multiple hand gestures need to be applied. Hence, this computation method is valid even when the number of combined synergy set exceeds the number of measured EMG signals. The HALS decomposes the normalized quasi-tension as follows:

(1) $$\begin{array}{c} \left[E\right]\;=\left[M\right]{\left[S\right]}^{\text{T}}, \end{array}$$

where *E* is the normalized quasi-tension signals in an *m* by *n* matrix with *m* being the number of time series and *n* the number of EMG channel inputs; *S*=[*s*_1_,…, *s*_*j*_] is the synergy set, where *j* is the number of synergies and *s*_*j*_=[*c*_1_,…*c*_*n*_]^T^ representing a single set of synergy, where *c*_*n*_ is the coactivation coefficient of EMG *n*; and furthermore, *M* is the coactivation coefficients of the synergy in *m* by *j* matrix:

(2) $$\begin{array}{c} \left[S_{k}\right]\leftarrow \left[S_{k}\right]+\frac{{\left(\left[E\right]{\left[M\right]}^{\text{T}}-\left[S\right]\left[M\right]{\left[M\right]}^{\text{T}}\right)}_{k}}{\left[M_{k}\right]{\left[M_{k}\right]}^{\text{T}}}, \end{array}$$

(3) $$\begin{array}{c} \left[M_{k}\right]\leftarrow {\left[M_{k}\right]}^{\text{T}}+\frac{{\left({\left[E\right]}^{\text{T}}\left[S\right]-{\left[M\right]}^{\text{T}}{\left[S\right]}^{\text{T}}\left[S\right]\right)}_{k}}{{\left[S_{k}\right]}^{\text{T}}\left[S_{k}\right]}. \end{array}$$

When the synergy model is derived, learning algorithm procedures are used to iterate (2) and (3) several times, where *k* (1, 2,…, *j*) denotes the label of synergies. The matrices *S* and *M* were computed using one set of single wrist motion data and single grip motion data.

In the analysis, wrist synergies with varying numbers from one to six were calculated from a wrist movement trial to confirm the validity number of synergies; thereafter, a single grip synergy was taken from a grip trial. More often than note, the variance account for (VAF) became the standard means of choosing the muscle synergy number [26–28]. In the same context, this study applies the number of synergies that matches over 0.9 VAF to all the subjects to ensure the synergy model consistency. The gains of the wrist motion synergy for the angles were derived using linear regression to compute the normalized sum and difference of the wrist angle (flexion-extension, radial-ulnar deviation) *θ*_*i*_ from the following equation:

(4) $$\begin{array}{c} \theta_{i}=a_{0,i}+a_{j,i}m_{j}+\cdots +\varepsilon , \end{array}$$

where *a*_0,*i*_ denotes the angle bias, *a*_*j*,*i*_*s* are the regression coefficients for each synergy coefficient *m*_*j*_, and *ε* denotes the random noise error. A combined synergy set with regression coefficients were used to estimate both grip motion and wrist motion task; hence, the synergies and gains from a combination of two trials were applied to all other tasks.

The computation of the wrist and grip synergies was conducted in two different ways. A facial image study showed that NMF learns the object in part-based representation [29]. In the case of grip motion, the muscles of all channels work together; therefore, multitrial-based muscle synergy was calculated in two ways: simultaneously from jointed wrist and grip trials (SLRM1) and separately per trial (SLRM2). The synergy sets were derived from comfortable maximum limit trials and grip trials.

### 2.6. Musculoskeletal Model (MSM)

The musculoskeletal model was used to compare the angle estimates of the synergy-based model. The MSM succeeded in estimating the joint angles of the elbow, wrist, and index finger with little influence from a change in wrist position [17]. The performances of SLRM and MSM were compared with each other to ascertain how good that of SLRM is; furthermore, the train set of MSM was taken from a comfortable maximum limit, which are the same trials used in SLRM. To optimize the MSM performance, MSM was derived from two different muscle numbers, namely, MSMS1 and MSMS2; the former used all measured muscles, while the latter used five wrist muscles. Kawase et al. constructed a one-degree-of-freedom model per joint [17]; to fit the model into this experiment, two wrist joint angles were converted as depicted in Figure 4.

#### 2.6.1. Statistical Analysis

An exhaustive cross-validation was used to test the performance of each model per subject, with indices used to estimate performance. The Pearson correlation coefficient (*r*) and normalized root-mean-square error (nRMSE) are defined as follows:

(5) $$\begin{array}{c} r=\frac{\Sigma_{i=1}^{n}\left(x_{i}-\bar{x}\right)\left(y_{i}-\bar{y}\right)}{\sqrt{\Sigma_{i=1}^{n}{\left(x_{i}-\bar{x}\right)}^{2}}\sqrt{\Sigma_{i=1}^{n}{\left(y_{i}-\bar{y}\right)}^{2}}},\\ \text{nRMSE}=\frac{1}{a}\sqrt{\frac{\Sigma_{i=1}^{n}{\left(x_{i}-y_{i}\right)}^{2}}{n}}, \end{array}$$

where *n* is the number of samples, *y* is a reference, *x* is an estimate, and *a* is defined as the normalization coefficient. nRMSE chooses *a* to be 90, the limit of the wrist angle range. All statistical analyses were conducted using *t* test2 function of MATLAB 2018b.

## 3. Results

### 3.1. Synergy Number Optimization

The reproducibility check of SLRM1 and SLRM2 in the different number of synergies was tested in VAF. The number of grip synergies was fixed to one, both in SLRM1 and SLRM2 to ensure that SLRM1 computed the synergy one more from the joint trials. SLRM1 with two wrist synergies had over 0.9 VAF on average (0.9342 ± 0.0245), and the minimum VAF of three wrist synergies was 0.9529. For SLRM2, three wrist synergies had over 0.9 VAF on average (0.9442 ± 0.0340), and the minimum VAF of four wrist synergies was 0.9647. To include all subjects, four wrist motion synergy numbers were chosen and used throughout the study.

### 3.2. Task 1: Wrist Motion Test

**Figure 5:**
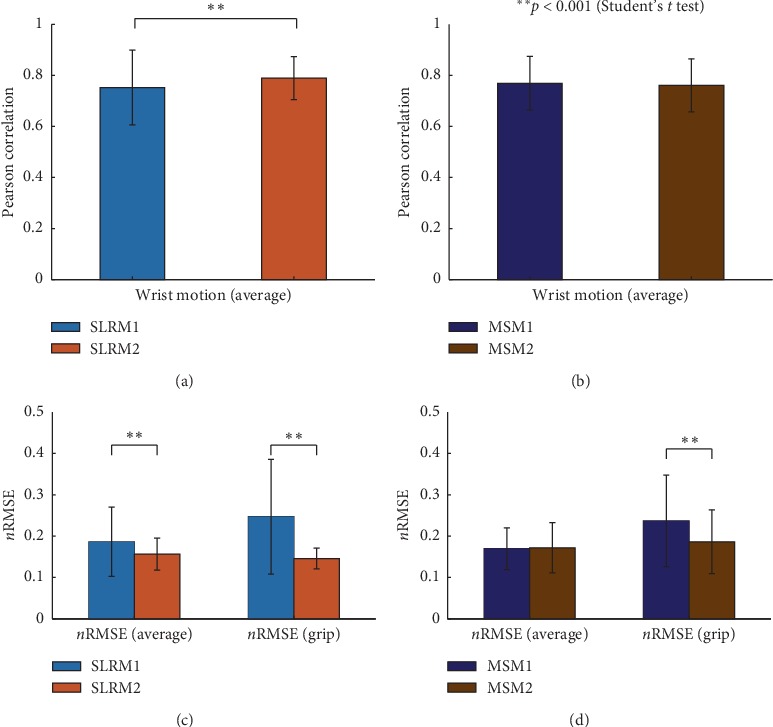
Model condition-related wrist joint movement estimation performance changes in the synergy-based linear regression model (SLRM) and musculoskeletal model (MSM) in terms of the Pearson correlation coefficient (*r*) and normalized root-mean-square error (nRMSE). (a) *r* of SLRM2 had statistically significant differences with SLRM1 (*p* < 0.001, Student's *t*-test). (b) There was no statistically significant change in *r* between MSM1 and MSM2. (c) nRMSE of SLRM1 and SLRM2 had statistically significant differences both in wrist average and grip motion with higher error in SLRM1. (d) There was no statistically significant change in the nRMSE for wrist motion trials between MSM1 and MSM2 while having a significant difference in grip. (*p* < 0.001, Student's *t*-test).

Figures 5(a) and 5(c) show the *r* and nRMSE values of SLRMs in wrist motion task. The *r* values of SLRM1 and SLRM2 were 0.7523 ± 0.1466 and 0.7891 ± 0.0844, respectively. For *n*RMSE, SLRM1 had 0.1864 ± 0.0835 in wrist motion and 0.2471 ± 0.1387 in grip motion, while SLRM2 had 0.1564 ± 0.0388 in wrist motion and 0.1458 ± 0.0251 in grip motion. The differences are statistically significant in both cases (*p* < 0.001, Student's *t*-test). From the results, SLRM2 was chosen as the representative SLRM model.

Similarly, Figures 5(b) and 5(d) show the *r* values and nRMSE values of the MSMs in wrist motion task. The *r* values of MSM1 and MSM2 were 0.7691 ± 0.1056 and 0.7608 ± 0.1037, respectively, which exhibits no statistical significance. For nRMSE, MSM1 had 0.1695 ± 0.0505 in wrist motion and 0.2368 ± 0.1107 in grip motion, while MSM2 had 0.1718 ± 0.0608 in wrist motion and 0.1864 ± 0.0770 in grip motion. The nRMSE of the grip motion implies a statistical significance (*p* < 0.001, Student's *t*-test). From the results, MSM2 was chosen as the representative MSM model.

**Figure 6:**
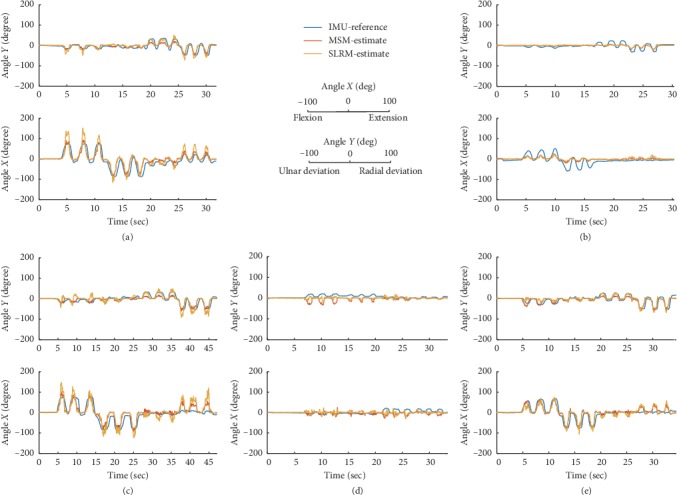
2D wrist joint angle estimation in 5 different trials. Angle *X* corresponds to flexion-extension dimension taking extension as positive. Angle *Y* corresponds to radial-ulnar deviation having radial deviation as positive. The blue-colored line represents the IMU-reference angle derived from two IMU sensors by differentiating relative orientation in the Euler angle. The red-colored estimate is a musculoskeletal model- (MSM-) based estimation having 5 input signals. Yellow-colored estimate stands for synergy-based linear regression model- (SLRM-) based estimation deriving synergy derived separately per trial. An example of (a) a comfortable maximum limit trial, (b) half of a comfortable maximum trial, (c) a stiffened movement trial, (d) a grip-trial having twelve times gripping, and (e) combined movement of a comfortable maximum limit with grip.

The time series of the wrist angle in two dimensions is shown in Figure 6. Subjects were asked to rotate their wrists in four directions. They were able to move freely at their own pace, moving in an inclined diagonal direction at different angles. Because of this tendency, both models appear to have the underlying assumption that a subject moved in a diagonal direction even if they performed a gradual movement, as shown in Figure 6(c).

The exact performances of SLRM and MSM in *r* and nRMSE are shown in Tables 2 and 3. Wrist motion performances *r* on average are 0.7891 ± 0.0844 in SLRM and 0.7608 ± 0.1037 in MSM, implying a statistically significant difference (*p* < 0.001, Student's *t*-test). Similarly, nRMSE also shows a significant difference between SLRM and MSM (*p* < 0.01, Student's *t*-test). This trend continued during wrist motion trials without a grip (comfortable maximum limit trial, comfortable half limit trial, and stiffened movement trial).

However, when grip motion was added, there was no statistically significant difference in *r*; however, differences were still apparent in nRMSE. The *r* of the comfortable maximum with grip trials were 0.7562 ± 0.0631 in SLRM and 0.7579 ± 0.0877 in MSM, and nRMSEs were 0.1654 ± 0.0.271 in SLRM and 0.1804 ± 0.0511 in MSM (*p* < 0.001, Student's *t*-test). Finally, in the grip trial, where *r* measurement was inappropriate because the wrist motion in the trial is just an indication of a perturbation, here, SLRM had 0.1458 ± 0.0251 and MSM had 0.1864 ± 0.0770 in nRMSE, implying a statistically significant difference (*p* < 0.001, Student's *t*-test).

In detail, the *r* values of the comfortable maximum limit trials were 0.8256 ± 0.0270 in SLRM and 0.7994 ± 0.0528 in MSM (*p* < 0.001, Student's *t*-test); the nRMSEs were 0.1539 ± 0.0178 in SLRM and 0.1713 ± 0.0343 in MSM (*p* < 0.001, Student's *t*-test). In comfortable half limit trials, the corresponding values were 0.7460 ± 0.0731 in SLRM and 0.6844 ± 0.1022 in MSM when measuring *r* values (*p* < 0.001, Student's *t*-test), and the nRMSEs were 0.1204 ± 0.0106 in SLRM and 0.1256 ± 0.0219 in MSM (*p* < 0.01, Student's *t*-test). The stiffened movement trials also had the same trend in *r* values, being 0.8406 ± 0.0344 in SLRM and 0.8142 ± 0.0589 in MSM (*p* < 0.001, Student's *t*-test); and, for nRMSEs, 0.1852 ± 0.0216 in SLRM and 0.2098 ± 0.0558 in MSM (*p* < 0.001, Student's *t*-test).

### 3.3. Task 2: Grip Motion Test

**Figure 7:**
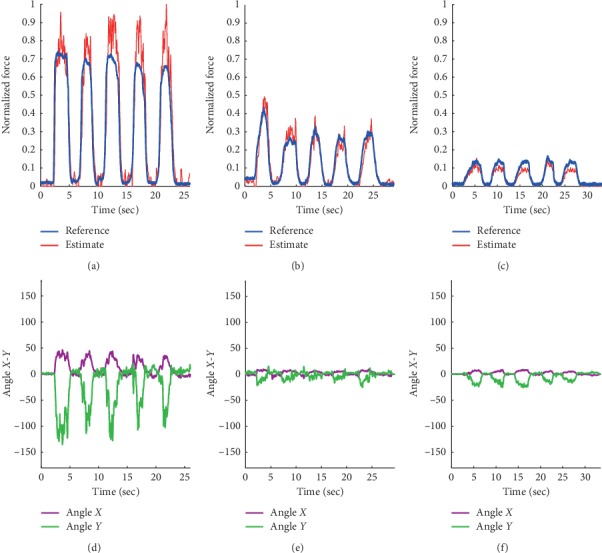
Time series estimation of grip task with ReachMAN robot. Time series for (a) the normalized max grip force reference and synergy-based linear regression (SLRM) grip force estimate, (b) the normalized half grip force reference and synergy-based linear regression (SLRM) grip force estimate, (c) the normalized quarter grip force reference and synergy-based linear regression (SLRM) grip force estimate, (d) wrist joint motion estimate from SLRM in the maximum grip force task, (e) wrist joint motion estimate from SLRM in half grip force task, and (f) wrist joint motion estimate from SLRM in quarter grip force task. Angle *X*-*Y* is the same axis in Figure 8.

Figure 7 shows the SLRM-based time series grip force estimate and resulting angle estimate perturbation. Subjects were constrained to a grip device during the task to ensure that there was no actual wrist motion during the entirety of task. Hence, wrist motion estimation during grip motion was checked for strong distortion in angle estimate. The results showed that during the gripping task, instability of the wrist angle estimation occurred in the presence of a strong force activation, as may be seen in Figure 7(d). For the half and quarter grip force task, angle estimation was less than 30°, as shown in Figures 7(e) and 7(f). The *r* value for grip force estimate and nRMSE of the *X*-*Y* angle estimate, compared with zero angle (no movement), were computed as indicated in Table 4. Sub8 data were omitted in this task because the EMG signal was saturated during the ADC converting process using NIDAQ (±5 voltage). The SLRM-based grip-force estimate from nine subjects was 0.8463 ± 0.0503 in *r* with 0.2559 ± 0.1397 nRMSE in wrist movement estimation.

## 4. Discussion

**Figure 9:**
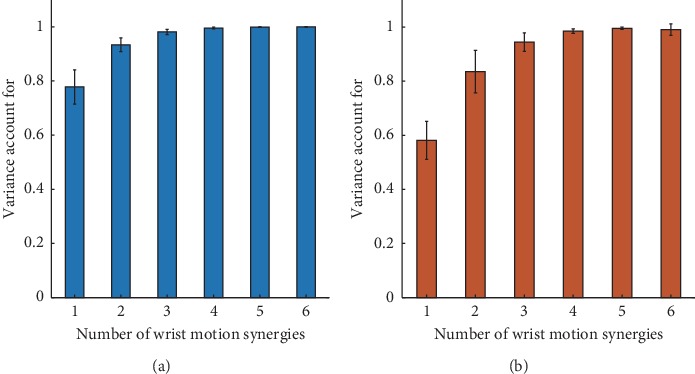
Two conditional SLRM-based R-square. (a) Variance account for with the different number of wrist motion synergies in SLRM1 (joint-trial-based synergy set) (b) Variance account for with the different number of wrist motion synergies in SLRM2 (separately derived synergy set).

**Figure 10:**
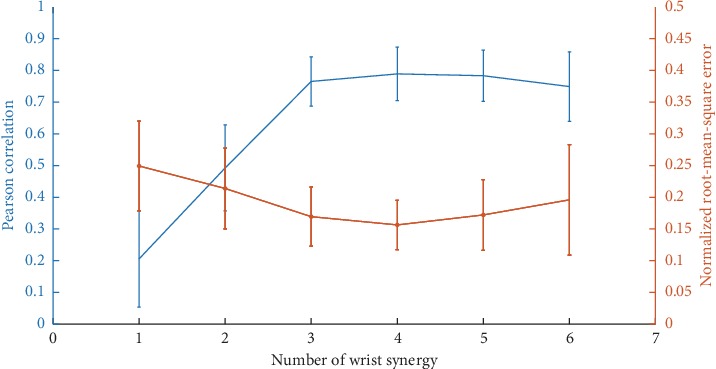
SLRM2-based two-dimensional wrist joint angle estimation performance with regard to the wrist synergy number.

This study tested both MSM and SLRM in two different conditions to optimize each model.  Figure 9 shows the VAF from SLRM1 and SLRM2 with the different number of wrist motion synergies. The VAF of SLRM1 converged in three wrist motion synergies and overfitted thereafter. This trend was the same as obtained in other studies whether in patients or healthy subjects [26–28]. For SLRM2, the VAF converged in four wrist motion synergies and overfitted up to five. There was also a decrease in VAF in SLRM2. d' Avella et al. suggested that when the number of extracted synergies is greater than the generator synergies combination, each additional synergy captures an equal amount of noise-generated variation [30]. In that sense, fifth and sixth muscle synergies are noise-derived synergies. This study measured five wrist motion muscles and two finger motion muscles; thus, the sixth muscle synergy in SLRM2 formulated synergy set with finger motion muscles, which made reproducibility impossible. Such noise-derived synergies contaminated the estimation performance. The wrist joint angle estimation performance of SLRM2 with varying numbers of wrist synergies are shown in Figure 10. The highest performance was obtained in four wrist motion synergies; the additional number of synergies deteriorates the estimation performance.

**Figure 11:**
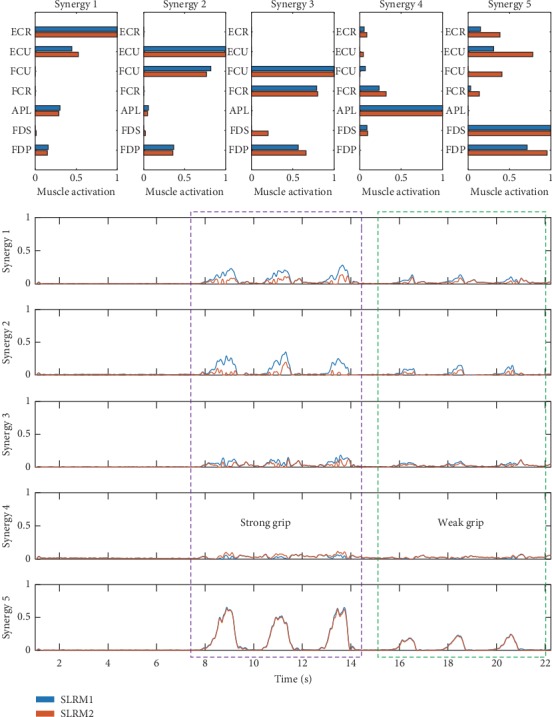
Time series synergy coefficient data with corresponding synergy matrix in the synergy-based linear regression model in the grip task. Synergies one to four represent wrist synergy, and synergy five represents grip synergy. The purple-colored region represents three-time strong grip actions and the green-colored region represents three-time weak grip actions.

In the SLRM, wrist movement estimation performance showed statistical significance depending on the synergy extraction method both in *r* and nRMSE. This study aims to use NMF for prosthetic and interface purposes; therefore, synergies were modulated per subject and trial. Separate synergy sets preserve multi-EMG coactivation in the grip synergy, which enables synergy to cluster movement type. In a joint-trial-based synergy set, the NMF divided multi-EMG signals into several part-based groups, resulting in tying the grip synergy to be a combination of EMG signals not used in other synergies. Therefore, the joint-trial-based synergy set could not discriminate movement type. This tendency can be seen in Figure 11. Synergy 5 had the most distinctive shape, representing grip synergy. In a time-domain reaction, SLRM1 responded to grip activation regardless of grip force. In SLRM2, it also increased other synergy coefficients albeit, relatively, by a small amount when a strong grip was assumed. This result could support extracting a unique synergy for each motion [31].

The FDS and FDP are flexor muscles of the fingers from an anatomical point of view. Those two muscles are separated by synergy analysis, which can be observed from Synergy 5, as shown in Figure 10. For the wrist motion with and without grip task, the other five muscles are appropriate to estimate joint angles. For the MSM, the minimum number of muscles exhibited good performance.

The estimation performance between SLRM and MSM had a statistically significant difference in the entire wrist-only movement task both in *r* and nRMSE values; however, there were no other *r* values in wrist motion with grip. The results show the robustness of the wrist movements with respect to finger movement of the MSM, which is the same as those obtained by Kawase et al.'s experiment [17]. The estimation performance of SLRM also showed comparable performance with previous results in the literature on trajectory [6] and joint force [9] estimation. The advantage of SLRM is that it estimates not only continuous wrist movements but also complex movements in addition to grip motion. In this experiment, we confirmed that the estimation performance of SLRM for complex motion was equivalent to MSM performance.

Within the wrist-only motion trials, both SLRM and MSM had the lowest performance in comfortable half performance. This is most likely due to the nonlinearity between EMG signals and arm motion or contamination of movement artifact and baseline noise to the EMG (representatively, Sub3). Therefore, the nonlinear regression techniques used in the previous studies [6, 9] could be comparable with the synergy-based model having an alternative to using linear regression.

The linear envelope filtering used in the EMG signal analysis was proven to have highly correlated signals with joint torque induced by the target muscle [21]. Grip synergy, which is a coactivation of these filtered EMG signals, also showed a high grip force estimation performance of 0.8463 ± 0.0503 without resort to further conversion or regression techniques. However, in the strong grip trial, it was confirmed that the wrist angle estimation was distorted by gripping EMG signals. This strong grip distortion indicates the necessity to investigate the limits of the SLRM in grip force estimation. Within the current experiment results, it is difficult to determine whether each subject distorted the wrist angle estimation with a similar absolute force level or with a specific ratio of the maximum force.

## 5. Conclusions

In this study, we explored a model for estimating wrist joint angle with and without grip action. In the first task, in which we examined wrist angle estimation of SLRM and MSM, the SLRM exhibited a relatively higher performance in wrist motion. In the grip task, SLRM showed robustness in angle estimation when the grip force is half or a quarter of its maximum force level. In addition, SLRM can provide the extent of grip force exerted in the center position with little perturbation. These characteristics of SLRM are useful for combined wrist and grip action; however, obtaining limiting grip force required to crash the wrist angle estimation, and vice versa, was beyond the scope of this study. Further studies are required to obtain the simultaneous estimation of both parameters necessary for daily usage.

## Figures and Tables

**Figure 8:**
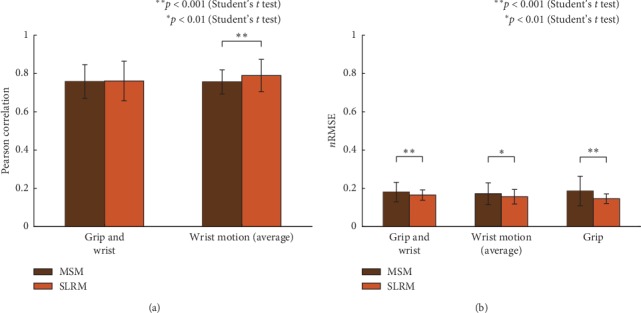
Trial-based wrist joint movement estimation performance changes in the synergy-based linear regression model (SLRM) and musculoskeletal model (MSM) in terms of the Pearson correlation coefficient (*r*) and normalized root-mean-square error (nRMSE). (a) SLRM and MSM had no statistically significant difference in wrist motion with grip, while wrist motion average had a statistically significant difference between the models (*p* < 0.001, Student's *t*-test). (b) nRMSE between SLRM and MSM had a statistically significant difference in every trial.

**Table 1:** Forearm muscle with channel number.

Muscle position	
Ch. 1	Extensor carpi radialis (ECR)
Ch. 2	Extensor carpi ulnaris (ECU)
Ch. 3	Flexor carpi ulnaris (FCU)
Ch. 4	Flexor carpi radialis (FCR)
Ch. 5	Abductor pollicis longus (APL)
Ch. 6	Flexor digitorum superficialis (FDS)
Ch. 7	Flexor digitorum profundus (FDP)

**Table 2:** MSM performance indicator per subjects and trial.

Sub	Data type	Comfortable max		Comfortable half		Stiffened movement		Grip and motion		Wrist motion (average)		Grip
		*r*	nRMSE	*r*	nRMSE	*r*	nRMSE	*r*	nRMSE	*r*	nRMSE	nRMSE
Sub1	*Mean* *SD*	0.8542 0.0218	0.1544 0.0167	0.7297 0.0725	0.1256 0.0123	0.8818 0.0156	0.1882 0.0348	0.8092 0.0661	0.1742 0.0315	0.8155 0.0805	0.1612 0.0371	0.1287 0.0214

Sub2	*Mean* *SD*	0.8527 0.0286	0.1024 0.0140	0.7626 0.0478	0.0719 0.0103	0.8362 0.0361	0.1666 0.0161	0.7300 0.1813	0.0971 0.0334	0.7902 0.1107	0.1101 0.0429	0.1468 0.0279

Sub3	*Mean* *SD*	0.6816 0.0475	0.1699 0.0214	0.5557 0.1039	0.1291 0.0195	0.7094 0.0590	0.2084 0.0322	0.6722 0.0616	0.1704 0.0220	0.6523 0.0968	0.1694 0.0407	0.1400 0.0608

Sub4	*Mean* *SD*	0.8261 0.0503	0.2113 0.0371	0.7196 0.0587	0.1196 0.0185	0.8178 0.0603	0.2593 0.0415	0.8278 0.0431	0.1820 0.0235	0.7952 0.0746	0.1914 0.0646	0.0975 0.0594

Sub5	*Mean* *SD*	0.8156 0.0835	0.1451 0.0342	0.6870 0.1324	0.1091 0.0163	0.8174 0.0526	0.2170 0.0422	0.7497 0.0604	0.2104 0.0401	0.7630 0.1019	0.1727 0.0581	0.3005 0.1109

Sub6	*Mean* *SD*	0.8364 0.0273	0.1998 0.0281	0.6855 0.1028	0.1940 0.0140	0.8003 0.0651	0.2613 0.0351	0.8055 0.0447	0.1934 0.0271	0.7770 0.0928	0.2132 0.0425	0.1741 0.0479

Sub7	*Mean* *SD*	0.6775 0.0697	0.2235 0.0205	0.4702 0.1268	0.1338 0.0113	0.6994 0.1093	0.2258 0.0370	0.6742 0.0790	0.1793 0.0243	0.6261 0.1386	0.1876 0.0480	0.5055 0.1803

Sub8	*Mean* *SD*	0.8078 0.0613	0.1333 0.0278	0.6616 0.0992	0.1104 0.0150	0.8478 0.0405	0.1524 0.0214	0.7541 0.0474	0.1578 0.0293	0.7642 0.1070	0.1389 0.0330	0.4311 0.0778

Sub9	*Mean* *SD*	0.8688 0.0443	0.1823 0.0298	0.8637 0.0420	0.1260 0.0096	0.8807 0.0256	0.1801 0.0227	0.8537 0.0407	0.1549 0.0210	0.8665 0.0425	0.1588 0.0315	0.3206 0.1702

Sub10	*Mean* *SD*	0.7728 0.0302	0.1915 0.0501	0.7087 0.0927	0.1361 0.0436	0.8517 0.0363	0.2392 0.1044	0.7024 0.1091	0.2844 0.1058	0.7576 0.1057	0.2147 0.1006	0.1750 0.0817

*Mean*	*Mean* *SD*	0.7994 0.0528	0.1713 0.0343	0.6844 0.1022	0.1256 0.0219	0.8142 0.0589	0.2098 0.0558	0.7579 0.0877	0.1804 0.0511	0.7608 0.1037	0.1718 0.0568	0.1864 0.0770

**Table 3:** SLRM performance indicator per subjects and trial.

Sub	Data type	Comfortable max		Comfortable half		Stiffened movement		Grip and motion		Wrist motion (average)		Grip
		*r*	nRMSE	*r*	nRMSE	*r*	nRMSE	*r*	nRMSE	*r*	nRMSE	nRMSE
Sub1	*MeanSD*	0.8847 0.0173	0.1368 0.0137	0.7850 0.0508	0.1155 0.0120	0.8618 0.0269	0.1738 0.0291	0.8625 0.0207	0.1313 0.0158	0.8452 0.0527	0.1396 0.0299	0.1121 0.0162

Sub2	*Mean SD*	0.8868 0.0200	0.0925 0.0148	0.7801 0.0251	0.0640 0.0073	0.8778 0.0391	0.1425 0.0166	0.7860 0.1166	0.0831 0.0184	0.8277 0.0867	0.0958 0.0339	0.1207 0.0098

Sub3	*Mean SD*	0.6643 0.0215	0.1658 0.0129	0.5037 0.0865	0.1205 0.0104	0.7749 0.0291	0.1704 0.0098	0.4568 0.0691	0.1750 0.0128	0.5941 0.1457	0.1572 0.0274	0.0620 0.0088

Sub4	*Mean SD*	0.8430 0.0178	0.1858 0.0176	0.7368 0.0661	0.1355 0.0166	0.8460 0.0208	0.2094 0.0239	0.7756 0.0305	0.1769 0.0103	0.7965 0.0654	0.1761 0.0341	0.1157 0.0189

Sub5	*Mean SD*	0.8368 0.0291	0.1444 0.0166	0.7698 0.0335	0.1001 0.0069	0.8321 0.0380	0.2390 0.0238	0.7598 0.0467	0.2403 0.0425	0.7963 0.0603	0.1843 0.0727	0.0888 0.0191

Sub6	*Mean SD*	0.8233 0.0266	0.1997 0.0179	0.7483 0.0848	0.1935 0.0074	0.8546 0.0228	0.2118 0.0155	0.8364 0.0216	0.1783 0.0169	0.8149 0.0665	0.1955 0.0215	0.2236 0.0432

Sub7	*Mean SD*	0.8009 0.0256	0.1622 0.0068	0.7162 0.1374	0.1328 0.0076	0.7955 0.0448	0.1767 0.0178	0.6393 0.0997	0.1762 0.0251	0.7323 0.1135	0.1620 0.0272	0.1653 0.0285

Sub8	*Mean SD*	0.8573 0.0243	0.1155 0.0115	0.8210 0.0541	0.0873 0.0133	0.8571 0.0320	0.1523 0.0193	0.7960 0.0454	0.1474 0.0316	0.8306 0.0547	0.1266 0.0358	0.2876 0.0503

Sub9	*Mean SD*	0.8417 0.0249	0.1788 0.0225	0.8240 0.0324	0.1380 0.0033	0.8316 0.0309	0.1901 0.0205	0.8196 0.0402	0.1606 0.0158	0.8281 0.0369	0.1658 0.0271	0.1037 0.0233

Sub10	*Mean SD*	0.8170 0.0329	0.1576 0.0227	0.7754 0.0232	0.1162 0.0049	0.8745 0.0250	0.1863 0.0270	0.8302 0.0265	0.1848 0.0363	0.8249 0.0618	0.1616 0.0417	0.1969 0.0299

*Mean*	*Mean SD*	0.8256 0.0270	0.1539 0.0178	0.7460 0.0731	0.1204 0.0106	0.8406 0.0344	0.1852 0.0216	0.7562 0.0631	0.1654 0.0271	0.7891 0.0844	0.1564 0.0382	0.1458 0.0251

**Table 4:** SLRM grip force estimate per subjects with corresponding wrist movement estimate perturbation.

Sub	Data type	Grip force estimate	Angle *X*-*Y* (average)
		*R*	nRMSE
Sub1	*Mean* *SD*	0.8408 0.0206	0.3068 0.0748

Sub2	*Mean* *SD*	0.7164 0.0579	0.4527 0.1922

Sub3	*Mean* *SD*	0.7909 0.1235	0.0925 0.0760

Sub4	*Mean* *SD*	0.8929 0.0184	0.1980 0.0370

Sub5	*Mean* *SD*	0.8682 0.0416	0.3162 0.1192

Sub6	*Mean* *SD*	0.9526 0.0098	0.2749 0.1652

Sub7	*Mean* *SD*	0.9488 0.0132	0.2001 0.1733

Sub8	*Mean* *SD*	—	—

Sub9	*Mean* *SD*	0.8588 0.0318	0.2436 0.0257

Sub10	*Mean* *SD*	0.7471 0.0198	0.2180 0.2319

*Mean*	*Mean* *SD*	0.8463 0.0503	0.2559 0.1397

## Data Availability

The experimental results from the individual participants are provided as a table within the article. Data of the raw EMG, IMU, and force sensors are available upon request to the corresponding author.
